# The prevalence and moderating factors of sleep disturbances in people living with HIV: a systematic review and meta-analysis

**DOI:** 10.1038/s41598-024-65713-x

**Published:** 2024-06-27

**Authors:** Suonaa Lee, Jae Won Oh, Kyung Mee Park, Jin Young Ahn, San Lee, Eun Lee

**Affiliations:** 1 Department of Psychiatry and the Institute of Behavioral Science in Medicine, Yonsei University College of Medicine, 50-1 Yonsei-ro, Seodaemun-gu, Seoul, 03722 Republic of Korea; 2 Department of Psychology, The University of Utah Asia Campus, Incheon, Republic of Korea; 3 Department of Hospital Medicine, Yongin Severance Hospital, Yonsei University College of Medicine, Yongin, Republic of Korea; 4 Division of Infectious Diseases, Department of Internal Medicine and AIDS Research Institute, Yonsei University College of Medicine, Seoul, Republic of Korea; 5 Department of Psychiatry, Yongin Severance Hospital, Yonsei University College of Medicine, Yongin, Republic of Korea; 6 Institute for Innovation in Digital Healthcare, Yonsei University, Seoul, Republic of Korea

**Keywords:** Diseases, Medical research, Risk factors

## Abstract

This systematic review and meta-analysis aimed to investigate the prevalence of self-reported sleep disturbances in people living with HIV considering the effects of age, depression, anxiety, CD4 cell counts, time since HIV diagnosis, study region, and the instruments used to measure sleep disturbances. We searched PubMed, PsycINFO, and EMBASE to include eligible articles. In this meta-analysis of 43 studies, the pooled prevalence of self-reported sleep disturbances was 52.29% (95% confidence interval 47.69–56.87). The subgroup analyses revealed that variations in the sleep measurements and study region significantly contributed to the observed heterogeneity. In the meta-regression analyses, higher proportions of participants with depression or anxiety and longer times since HIV diagnosis were significantly associated with a higher prevalence of self-reported sleep disturbances after adjusting for mean age. Our findings emphasise the substantial burden of sleep disturbances in people living with HIV and identified comorbid depression and anxiety and the time since HIV diagnosis as significant moderators. These results underscore the importance of considering these factors when designing tailored screening programmes for high-risk patients and implementing early interventions to prevent and mitigate sleep disturbances in people living with HIV.

## Introduction

Over the past two decades, significant progress has been made in understanding, preventing, and treating HIV, leading to a substantial decrease in its incidence worldwide and remarkable improvements in the life expectancy of individuals undergoing antiretroviral therapy (ART)^1^. This change has given rise to a burgeoning emphasis in both research and medical care on enhancing the quality of life for people living with HIV (PLWH)^2,3^.

Sleep disturbances appear to be a common complaint among PLWH and can occur at any time following HIV diagnosis^4–7^. Sleep disturbances have been known to significantly impair quality of life and interfere with adherence to prescribed treatments for PLWH. Previous studies have indicated that PLWH experience insomnia and other sleep-related difficulties, with prevalence rates ranging from 40 to 100% depending on the definition and methodology, in contrast to the general population’s range of 13–30%^8,9^. However, despite their high prevalence in PLWH, sleep disturbances are frequently underrecognised and untreated in this population^10^.

Although the mechanism of sleep disturbance in PLWH has not been definitively elucidated, the potential contributing factors include the immune response to the virus, subsequent neurotoxic effects within the central nervous system^11^, opportunistic infections in the central nervous system^12^, psychological factors, such as depression and anxiety^13^, a lack of social support^14^, and the side effects of antiretroviral drugs^6,15^. Despite the uncertainty surrounding the aetiology, sleep disturbances have become clinically significant in PLWH as the focus in HIV care has shifted from mere patient survival to managing comorbidities. For example, sleep disturbances in this population are related to impaired daytime functioning and psychiatric symptoms, including depression and anxiety^16^, and contribute to comorbid metabolic dysregulation^17^. Furthermore, sleep disturbances can reduce adherence to ART, thereby accelerating disease progression^18^.

Given the concurrent presence of sleep disturbances and HIV infection, clinical management becomes more intricate^14^. Therefore, understanding the moderating factors of sleep disturbances in PLWH is crucial for maintaining a good quality of life and reducing comorbidities. However, studies exploring the potential moderators of sleep disturbances in PLWH remain scarce. Moreover, the previous meta-analysis estimating the prevalence of sleep disturbances in PLWH did not consider depression and anxiety as moderators^9^. To address this gap, we conducted an updated systematic review and meta-analysis to assess the prevalence of self-reported sleep disturbances in PLWH considering several moderators: age, depression, anxiety, CD4 cell counts, time since HIV diagnosis, the continent where the study was performed, and the instruments used to measure sleep disturbance.

## Results

### Study flow

**Figure 1:**
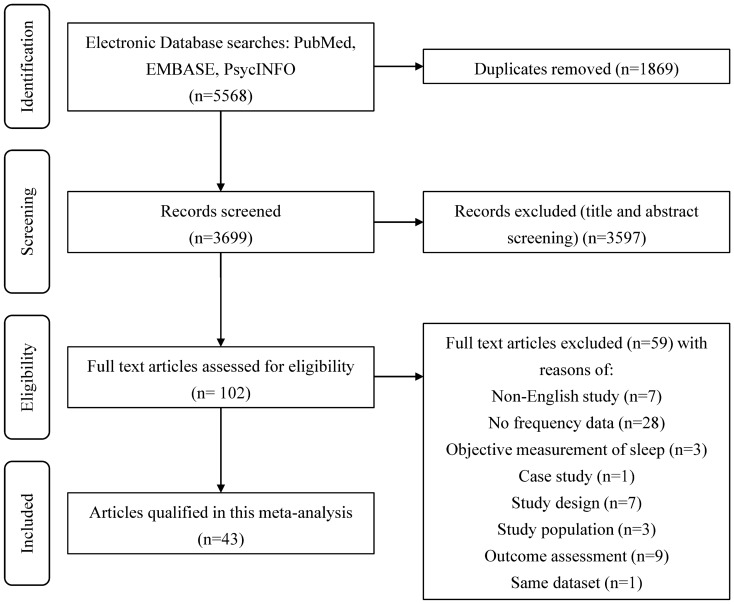
PRISMA flow diagram of study selection.

A total of 5568 studies were initially retrieved from the three databases. After removing duplicates (1869 studies), the titles and abstracts of 3699 articles were screened. Subsequently, 102 studies were deemed potentially eligible and underwent a full-text review. A total of 43 studies were finally selected for the meta-analysis (see Fig. 1) after 59 studies were removed for not meeting the inclusion criteria.

### Study characteristics

The 43 studies were published between 1998 and 2023 and included a total of 28,480 participants with HIV infection. The sample sizes ranged from 25 to 13,700 participants. Of the 43 studies, 17 were conducted in North America (all in the United States), 11 were conducted in Asia (5 in China, 4 in Iran, 2 in Indonesia), 7 were conducted in Africa (4 in Ethiopia, 2 in Nigeria, 1 in the Republic of South Africa), 6 were conducted in Europe (2 in the United Kingdom, 2 in France, 1 in Greece, 1 in Romania), and 2 were conducted in South America (both in Brazil). Most of the studies were cross-sectional (k = 40). Thirty-four studies used the PSQI, one of which employed a 2-item PSQI. Nearly all the studies that used the full-item PSQI used a cut-off value of 5 to distinguish poor sleepers from good sleepers; one study instead used a cut-off value of 10^19^. Eighteen studies reported the percentage of comorbid depression, and 11 studies reported the percentage of comorbid anxiety. Twenty-eight studies reported the time since HIV diagnosis. Detailed information for each study is presented in Table 1. The study quality assessment scores ranged from 5 to 9; all studies were evaluated to have moderate or high quality (see Supplementary Table S1).

**Table 1:** Summary of the characteristics of the included studies.

Author	Year	Study design	Study region	Mean age ± SD (years)	Male (%)	Sample size (HIV +)	Prevalence (%)	Sleep measurement and cut-off score	Depression	Anxiety	CD4 count (cells/mm^3^)	Mean time since HIV diagnosis (years)
Rubinstein et al.^57^	1998	Cross-sectional	North America	39.9 (8.3)	69	115	73	PSQI (> 5)	HADS-D (> 10) 32%	HADS-A (> 10) 55%	< 200 (49%) 200–500 (32%) > 500 (19%)	NR
Cruess et al.^58^	2003	Cross-sectional	North America	38.8 (8.5)	71.9	57	61.4	PSQI (> 5)	NR	NR	474.4 (242.2)	NR
Robbins et al.^20^	2004	Cross-sectional	North America	39.9 (range 24–63)	46.8	79	100	PSQI (≥ 5)	NR	NR	NR	NR
Phillips et al.^59^	2005	Cross-sectional	North America	34.8 (6.8)	0	144	66	PSQI (> 5)	NR	NR	NR	< 1 (2.2%) 1 to < 5 (42.3%) 5 to < 10 (40.9%) ≥ 10 (14.6%)
Junqueira et al.^60^	2008	Cross-sectional	South America	35.4 (range 10–51)	0	30	86.7	PSQI (> 5)	BDI (moderate to severe) 40%	BAI (moderate to severe) 53.3%	NR	NR
Marion et al.^61^	2009	Cross-sectional	North America	30.7 (8.6)	0	60	50	PSQI (≥ 5)	CES-D 13.3 (10.9)	NR	446.4 (286.3)	NR
Salahuddin et al.^62^	2009	Cross-sectional	North America	43.3 (7.5)	65.6	128	80	PSQI (> 5)	NR	NR	NR	10.3
Crum-Cianflone et al.^63^	2012	Cross-sectional	North America	35.9 (8.6)	95.3	193	46.1	PSQI (> 5)	BDI-II (≥ 20) 23.3%	NR	586.8 (230.1)	7.2
Lee et al.^64^	2012	Cross-sectional	North America	45 (8.4)	67	290	65	PSQI (> 5)	NR	NR	450.0 (267.0)	12.1
Chen et al.^29^	2013	Cross-sectional	North America	39.3 (7.1)	0	107	42.5	GSDS (> 3)	NR	NR	232.0 (no SD)	4.76
Seay et al.^65^	2013	Cross-sectional	North America	38.3 (7.8)	0	139	59	PSQI (> 5)	NR	NR	484.5 (303.0)	7.7
Dabaghzadeh et al.^12^	2013	Cross-sectional	Asia	36.9 (9.8)	71.2	59	47.5	PSQI (> 5)	HDRS (≥ 14) 42.4%	HARS (≥ 18) 3.4%	157.9 (117.2)	NR
Gamaldo et al.^66^	2013	Longitudinal	North America	50.3 (6.1)	100	25	78.3	PSQI (> 5)	BDI Mean: 8.4 SD: 10.2	STAI state 38.3 (10.5) STAI trait 37.7 (25.1)	NR	NR
Oshinaike et al.^5^	2014	Cross-sectional	Africa	38.9 (10.3)	29.3	300	59.3	PSQI (≥ 5)	NR	NR	≤ 200 (32.3%) > 200 (67.7%)	2.3
Jabbari et al.^67^	2015	Cross-sectional	Asia	35.9 (7.8)	67	150	55.3	PSQI (> 5)	HADS 7* (IQR 7)	NR	NR	NR
Downing et al.^21^	2016	Cross-sectional	North America	39.6 (12.3)	100	13,700	33.2	PSQI (2 items from PSQI)	PHQ-2 (≥ 3) 23.3%	GAD-2 (≥ 3) 22.4%	≤ 200 (5.9%) 201–349 (9.1%) 350–499 (15.3%) ≥ 500 (48.5%) Don’t know or missing (21.2%)	NR
Allavena et al.^6^	2016	Cross-sectional	Europe	47 (2.1)	73.5	1036	47.0	PSQI (> 5)	BDI-II (≥ 19) 19.7%	NR	607.3 (259.7)	12.8
Byun et al.^68^	2016	Cross-sectional	North America	44.8 (8.5)	63	268	63	PSQI (> 5)	NR	NR	< 200 (17%)	NR
Huang et al.^40^	2017	Cross-sectional	Asia	37.6 (11.7)	78.1	4103	43.1	PSQI (> 5)	HADS 5* (IQR 2–8)	HADS 5* (IQR 2–9)	< 50 (3.1%) 50–200 (12.3%) 200–500 (52.6%) ≥ 500 (31.0%) Missing (0.9%)	2.7
Arbune et al.^69^	2017	Cross-sectional	Europe	24–29 (66%) 30–39 (22%) 40–50 (12%)	54	102	42	PSQI (> 5)	NR	NR	< 200 (16%) 200–500 (28%) > 500 (56%)	> 5 (81%)
Ren et al.^70^	2018	Cross-sectional	Asia	≤ 45(30.7%) > 45(36.2%)	95.78	237	32.07	Spigel scale (≥ 18)	BDC (≥ 11) 98.3%	Self-Rating Anxiety Scale (≥ 45) 15.2%	≤ 350 (52.7%) > 350 (47.3%)	≤ 5 (53.2%) > 5 (46.8%)
Redman et al.^71^	2018	Cross-sectional	Africa	42.7 (9.2)	21	139	61	PSQI (> 5)	BDI (> 17) 41%	NR	434. 7 (278.5)	6.8
Fekete et al.^37^	2018	Cross-sectional	North America	42.81 (11)	71.8	181	71.3	PSQI (> 5)	CES-D (≥ 19) 53.6%	NR	NR	11.7
Faraut et al.^72^	2018	Cross-sectional	Europe	47.7 (11.1)	72.3	425	68	PSQI (> 5)	BDI (≥ 11) 16%	NR	617.0 (264.5)	14.7
Ding et al.^73^	2018	Cross-sectional	Asia	52.4 (9.0)	73.8	244	18.9	Jenkins Sleep Scale	SDS 16.6 (4.8)	NR	≥ 200 cells/μL (84.0%)	3.1
Gutierrez et al.^10^	2019	Cross-sectional	North America	49.0 (9.0)	44	176	73.3	PSQI (> 5)	PHQ-9 (≥ 10) 48%	NR	588.7 (365.5)	12.2
Ning et al.^74^	2020	Cross-sectional	Asia	44.4 (14.6)	76	1469	24.1	PSQI (> 5)	SDS 14.0 (4.5)	NR	≥ 500 cells/μL (39.6%)	< 1 (29.2%)
Mahboobi et al.^75^	2020	Cross-sectional	Asia	39.8 (9.7)	67.8	298	70.4	PSQI (≥ 5)	DASS (cut-off NR) 56.7%	DASS (> 6) 51.7%	577.0 (301.6)	5.2
Abdu et al.^76^	2020	Cross-sectional	Africa	32.6 (8.5)	55.1	336	57.1	PSQI (> 5)	NR	NR	NR	≤ 7.8 (44.9%) > 7.8 (55.1%)
Pujasari et al.^77^	2020	Cross-sectional	Asia	34.9 (5.0)	78.5	200	33.5	ISI (> 10)	HADS 6.3 (3.2)	HADS 6.0 (3.8)	NR	NR
Bedaso et al.^48^	2020	Cross-sectional	Africa	38.2 (9.7)	40.6	389	57.6	PSQI (> 5)	HADS (≥ 8) 30.6%	HADS (≥ 8) 31.9%	< 200 (2.8%) 200–499 (30.6%) ≥ 500 (66.6%)	≤ 1 (19.3%) > 1 (80.7%)
D De Francesco et al.^78^	2021	Cross-sectional	Europe	57.0 (7.4)	86.6	342	22.9	ISI (≥ 15)	PHQ-9 (≥ 10) 12.4%	NR	648.0 (259.1)	17.7
Rogers et al.^30^	2021	Cross-sectional	North America	51.6 (8.5)	49.51	103	34.94	ISI (≥ 15)	PHQ-9 7.12 (5.83)	NR	511.0 (309.4)	NR
Kunisaki et al.^79^	2021	Cross-sectional	Europe	Older (> 50) PWH (range 56–65) Younger (18–50) PWH (range 40–50)	80.3	357	21.5	ISI (≥ 15)	NR	NR	Older PWH 597 (range 470–780) Younger PWH 610 (range 470–779)	NR
Pujasari et al.^80^	2021	Cross-sectional	Asia	34.9 (5.0)	78.5	200	33.5	ISI (> 10)	NR	NR	NR	6.9
Najafi et al.^81^	2021	Cross-sectional	Asia	40.0 (9.6)	68.8	304	72	PSQI (> 5)	DASS (≥ 13) 47.4%	DASS (≥ 13) 43.7%	577.1 (301.2)	5.2
Daubert et al.^82^	2022	Cohort	North America	NR	0	1123	51.6	PSQI (> 5)	NR	NR	690.3 (330.2)	NR
Legas et al.^83^	2022	Cross-sectional	Africa	18–30 (50.1%) 31–45 (49.9%)	0	411	39.4	PSQI (> 5)	NR	GAD-7 (≥ 10) 34.8%	NR	NR
Cunha et al.^19^	2022	Cross-sectional	South America	45.3 (13.4)	64.7	385	43.38	PSQI (> 10)	NR	NR	543.7 (322.2)	9.5
Chen et al.^84^	2022	Longitudinal	Asia	29.4 (7.5)	98.6	217	56.2	PSQI (> 5)	CES-D (≥ 10) 37.7%	NR	358.6 (214.5)	0.3
Awopeju et al.^85^	2022	Cross-sectional	Africa	44.5 (20.3)	20.1	401	22.9	PSQI (> 5)	NR	NR	626.1 (301.0)	5
Petrakis et al.^86^	2023	Cross-sectional	Europe	45.6 (11.8)	80.5	154	55.2	AIS (≥ 6)	HADS (> 8) 46.1%	HADS (> 8) 54.5%	667.3 (370.4)	7.3
GebreEyesus et al.^14^	2023	Cross-sectional	Africa	36.0 (6.5)	36.3	419	36	PSQI (> 5)	HADS (> 8) 37.2%	HADS (> 8) 30.5%	≥ 350 (60.9%) 200–350 (19.1%) < 200 (19.3%) Other (0.1%)	> 5 (63.5%) ≤ 5 (36.5%)

*AIS* Athens insomnia scale, *BAI* Beck anxiety inventory, *BDC* burns depression checklist, *BDI* Beck depression inventory, *CES-D* center for epidemiological studies depression scale, *DASS* depression anxiety stress scale, *GAD* generalised anxiety disorder, *HADS-A* hospital anxiety and depression scale anxiety subscale, *HADS-D* hospital anxiety and depression scale depression subscale, *HDRS* Hamilton Depression rating scale, *HIV* human immunodeficiency virus, *ISI* insomnia severity index, *IQR* interquartile range, *NR* not reported, *PHQ* patient health questionnaire, *PSQI* Pittsburgh sleep quality index, *PWH* persons with HIV, *SD* standard deviation, *SDS* Zung self-rating depression scale, *STAI* state–trait anxiety inventory.

*Median.

### Pooled prevalence of sleep disturbances among PLWH

**Figure 2:**
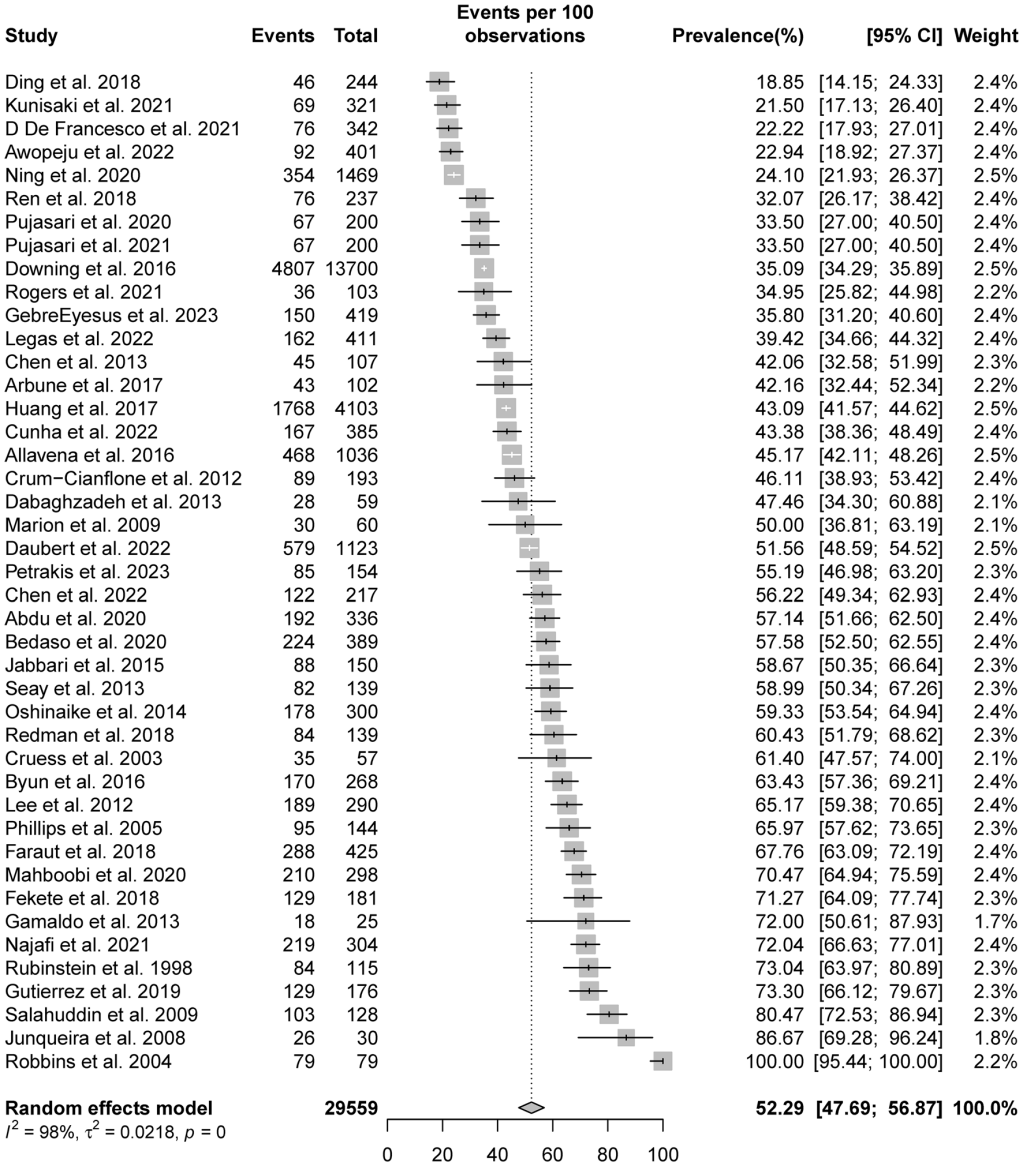
Forest plot of pooled prevalence of self-reported sleep disturbances in people living with HIV.

The overall estimated prevalence of self-reported sleep disturbances from the 43 studies was 52.29% (95% CI 47.69–56.87), although the studies showed high levels of heterogeneity (*I*^*2*^ = 97.9% p < 0.001). The prevalence in the included studies ranged from 18.9 to 100%. The details of the individual effect sizes and pooled effect are presented in Fig. 2. Given the significant variations in sample size and prevalence among the included studies, a sensitivity analysis was conducted to assess the impact of each study on the heterogeneity. The sensitivity analysis found that the prevalence estimates and heterogeneity did not significantly differ from the primary results (see Supplementary Fig. S3). After using the leave-one-out method to test the robustness of our meta-analysis, a Baujat plot was used to investigate which studies may have contributed to the high levels of heterogeneity detected (see Supplementary Fig. S4). The results show that the studies by Robbins et al.^20^ and Downing et al.^21^ had considerable influence on the overall heterogeneity levels. After excluding these two studies, the overall prevalence was 51.17% (95% CI 46.15–56.18).

### Assessment of sleep disturbances among PLWH by moderators

**Figure 3:**
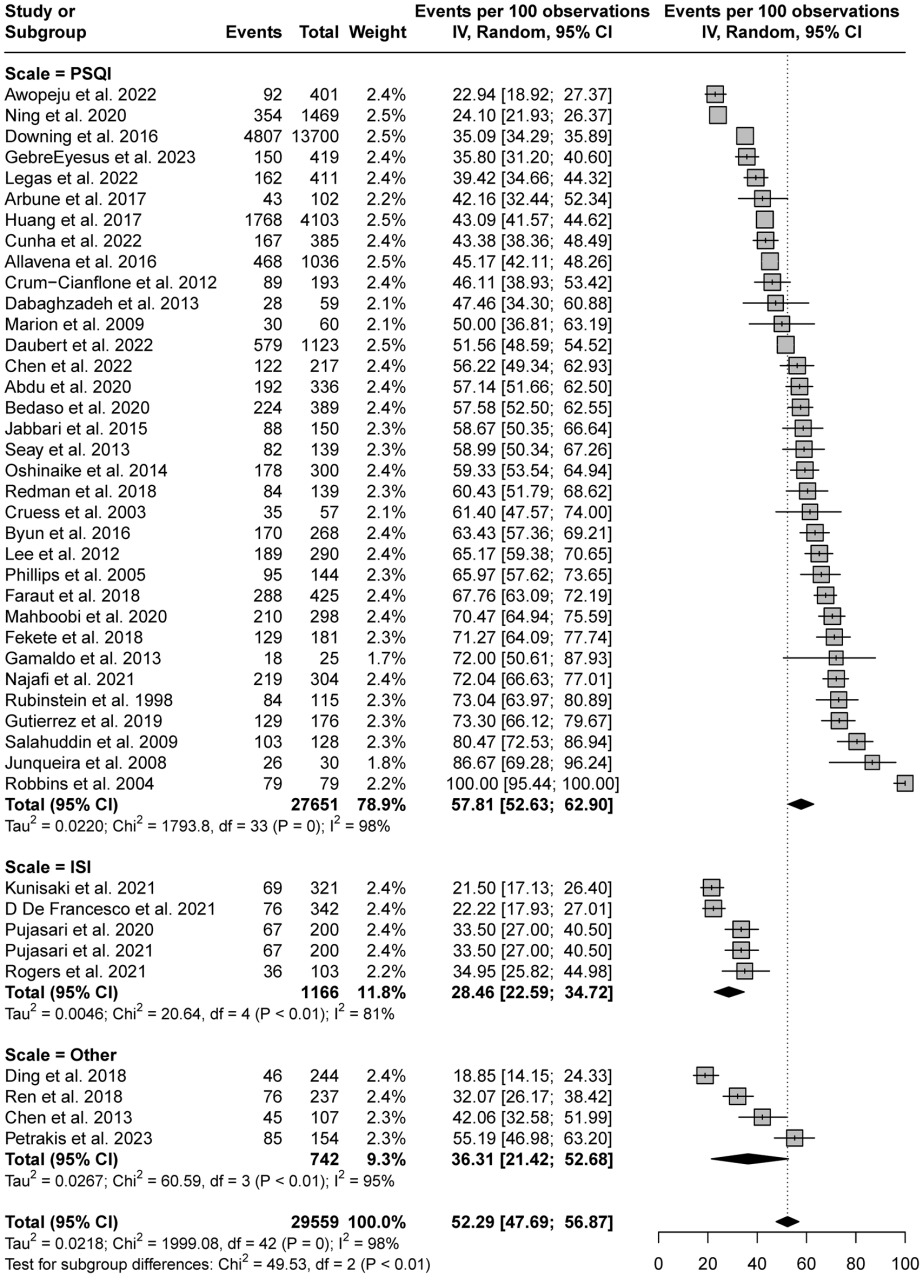
Subgroup analysis by sleep instruments.

Due to the high levels of heterogeneity, further subgroup analyses were performed to examine the prevalence of self-reported sleep disturbances based on the different measurements used in the studies. As shown in Fig. 3, the PSQI was the most frequently used measurement tool. The prevalence was significantly higher in studies using the PSQI (57.81%, 95% CI 52.63–62.90) compared with those using the ISI (28.46%, 95% CI 22.59–34.72) or other sleep instruments (36.31%, 95% CI 21.42–52.68).

**Figure 4:**
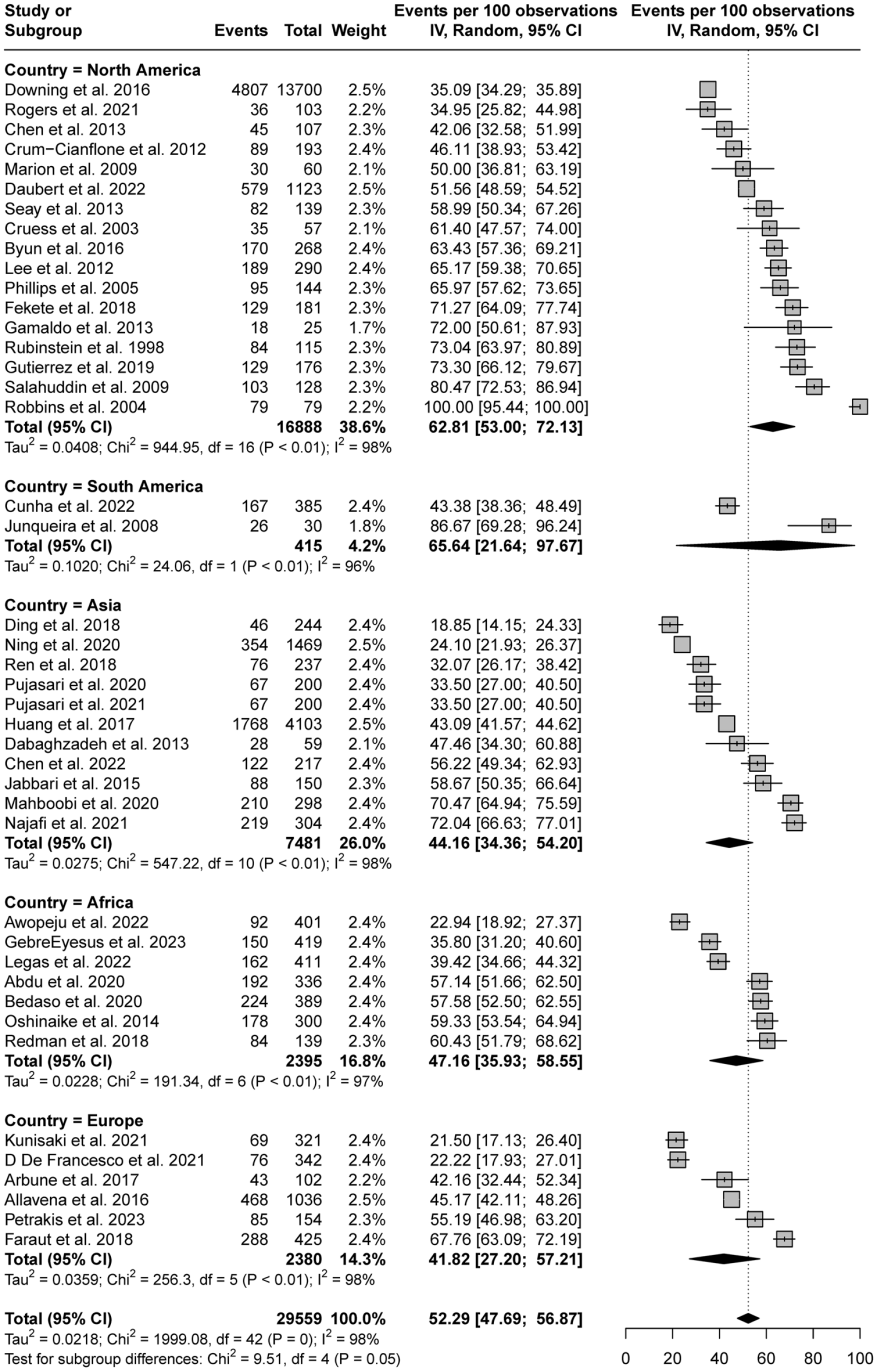
Subgroup analysis by continent where the study was performed.

Further subgroup analyses were conducted based on the continents where the studies were performed (Fig. 4). Significant differences were observed among the continents. The pooled estimates were 62.81% (95% CI 53.00–72.13) for North America, 65.64% (95% CI 21.64–97.67) for South America, 44.16% (95% CI  34.36–54.20) for Asia, 47.16% (95% CI 35.93–58.55) for Africa, and 41.82% (95% CI 27.20–57.21) for Europe.

When comparing studies published before 2015 with those published from 2015 onward, the group including papers published before 2015 (66.54%, 95% CI 57.09–75.38) showed a significantly higher prevalence of sleep disturbances compared to the group including papers published from 2015 onward (45.96%, 95% CI 41.13–50.83, p = 0.0002). When analysing 34 papers measured with PSQI, those published after 2015 demonstrated a significantly higher prevalence of sleep disturbances as well.

Additional meta-regression analyses were conducted to investigate the substantial heterogeneity across the studies. In the univariable meta-regression analyses, a higher proportion of participants with comorbid anxiety (*β* = 0.008, 95% CI 0.004–0.012, *k* = 11, *Q*_*M*_ = 17.44, *p* < 0.001, Supplementary Fig. S5) was significantly associated with a higher prevalence of self-reported sleep disturbances. The mean age, proportion of participants with comorbid depression, mean CD4 cell counts, and mean time since HIV diagnosis were not significantly associated with the prevalence of sleep disturbances. After controlling for mean age, a higher proportion of participants with depression (*β* = 0.007, 95% CI 0.002–0.012, *k* = 17, *Q*_*M*_ = 7.47, *p* = 0.024), longer time since HIV diagnosis (*β* = 0.023, 95% CI 0.002–0.043, *k* = 21, *Q*_*M*_ = 6.41, *p* = 0.041), and higher proportion of participants with anxiety (*β* = 0.009, 95% CI 0.003–0.015, *k* = 9, *Q*_*M*_ = 9.10, *p* = 0.011) were significantly associated with a higher prevalence of self-reported sleep disturbances. The mean CD4 cell count was not significantly associated with the prevalence of sleep disturbances, even after adjusting for age. Detailed results are reported in Table 2.

**Table 2:** Association between sleep disturbances and moderators.

**Variable**	Univariable analysis			Multivariable analysis^†^		
	Number of studies	Overall estimate (95% CI)	*p*-value	Number of studies	Overall estimate (95% CI)	*p*-value
Mean age (years)	38	− 0.006 (− 0.014, 0.002)	0.141			
Mean CD4 cell count (cells/mm^3^)	20	< − 0.001 (− 0.001, 0.001)	0.893	19	< 0.001 (− 0.001, 0.001)	0.432
Mean time since HIV diagnosis (years)	21	0.005 (− 0.012, 0.023)	0.541	21	0.023 (0.002, 0.043)	0.041
Proportion of depression (%)	18	0.001 (− 0.003,0.005)	0.574	17	0.007 (0.002, 0.012)	0.024
Proportion of anxiety (%)	11	0.008 (0.004, 0.012)	< 0.001	9	0.009 (0.003, 0.015)	0.011

^†^Each variable was adjusted for age.

### Publication bias

The funnel plot (Supplementary Fig. S1) and Egger’s test (*t* = 4.03, *p* < 0.001) indicated significant publication bias. The results of the trim-and-fill analysis show that at least 16 additional studies would be required for a symmetrical effect size distribution (Supplementary Fig. S2). After incorporating these effects, the pooled estimate of the prevalence of self-reported sleep disturbances was 38.88% (95% CI 34.24–43.61), which is significantly lower than the prevalence based on the primary result (52.29% [95% CI 47.69–56.87]). These results suggest that studies reporting a lower prevalence of self-reported sleep disturbances were less likely to be published compared with those reporting higher prevalence rates.

## Discussion

We performed this systematic review and meta-analysis to examine the prevalence of self-reported sleep disturbances in PLWH, building upon and extending the findings of a meta-analysis published in 2015^9^. We included 43 studies with a total of 28,480 participants. In our review, the pooled prevalence of sleep disturbances was 52.29% (95% CI 47.69–56.87), which is comparable to the prevalence of 58.0% reported in the previous meta-analysis^9^. These estimates are substantially higher than the reported estimate of 30% for the general population^22^. In the sensitivity analysis, the pooled prevalence remained robust at 51.17% (95% CI 46.15–56.18) after excluding two studies that potentially contributed to heterogeneity from the Baujat plot. The subgroup and meta-regression analyses revealed that the continent, comorbid depression rate, comorbid anxiety rate, time since HIV diagnosis, and instrument used to measure sleep disturbances were significant moderators. These findings enhance our understanding of the variability in the prevalence of sleep disturbances across the studies included in the meta-analysis.

The heterogeneity in the prevalence of sleep disturbances among PLWH can be attributed to the varying sleep measurement instruments in the included studies. In our review, the PSQI was the most frequently used measurement tool in studies collecting prevalence data, followed by the ISI. Consistent with previous meta-analyses of different populations^23–25^, we observed significant differences in the prevalence of sleep disturbances among PLWH in studies using different assessment instruments. The prevalence was significantly higher in the studies using the PSQI (57.80%, 95% CI 52.63–62.90) than in those using the ISI (28.46%, 95% CI 22.59–34.72) or other sleep instruments (36.31%, 95% CI 21.42–52.68). This discrepancy may be attributed to variations in the range of symptoms covered by each instrument. For example, the ISI evaluates all three major insomnia symptoms—difficulty initiating sleep, difficulty maintaining sleep, and early morning awakening^26^—whereas the PSQI assesses a broad range of sleep domains influencing overall sleep quality. Considering that the previous meta-analysis (although it focused on individuals other than HIV-infected patients) found no significant difference in sleep disturbance prevalence between the PSQI and ISI subgroups, which had comparable numbers of studies^27^, the observed discrepancy in prevalence between the PSQI and ISI subgroups in our study may be attributed to an uneven distribution of studies within the subgroups, which had 34 and 5 studies, respectively. In addition, the study by Robbins et al.^20^ reported a 100% prevalence of sleep disturbances in PLWH, which led to an overall higher prevalence rate in the studies using the PSQI. After excluding the study by Robbins et al., the prevalence of sleep disturbances decreased to 55.9% in the subgroup analysis.

Our findings show significant differences in the prevalence of sleep disturbances among the studied continents, with the highest prevalence observed in South America, followed by North America, Africa, Asia, and Europe. Given that the highest prevalence of depression in HIV-infected patients was reported in South America and the lowest was reported in Europe^28^, psychological burden may have influenced the prevalence of sleep disturbances. In addition, a previous epidemiologic study in the general population reported that women are more likely to report sleep disturbances than men^22^; thus, the relatively lower prevalence of sleep disturbances in Asia and Europe may have been influenced by the higher proportions of male participants in all the studies conducted in these regions. However, considering the sex disparities in the prevalence of HIV infection among continents, and noting that most of the studies included in this meta-analysis were conducted in North America with relatively fewer studies from Asia and Europe, further research regarding the impact of sex on sleep disturbances is needed. We also found that of the many studies conducted in South and North America, all but two^29,30^ used the PSQI to measure sleep disturbances. In light of our finding that the use of the PSQI was associated with a higher reported prevalence of sleep disturbances compared with other sleep instruments, methodological differences may have partially contributed to the geographical disparity in sleep disturbance rates.

We observed that the prevalence of sleep disturbances was significantly lower in studies published from 2015 onward compared to those published before 2015. Regarding integrase inhibitor-based ART has fewer reported sleep-related side effects^31^ compared to efavirenz^32^, which is known to increase sleep disturbances, this finding may reflect the increased use of integrase inhibitors-based ART instead of efavirenz. In addition, considering previous findings suggesting an association between stigma and sleep impairment^33^, it is plausible that the decreasing HIV-related stigma over time^34^ may have influenced the lower prevalence of sleep disturbance observed from 2015 onward.

We found that higher proportions of patients with anxiety or depressive symptoms were associated with higher estimates of sleep disturbance prevalence even after adjusting for mean age. Given the bidirectional relationship between insomnia and depression and anxiety—where sleep disturbances can either precede or be caused by depression and anxiety^16^—our findings suggest that effective treatment of either sleep disturbances or depression/anxiety may potentially resolve or prevent the other, thereby improving quality of life. Given concerns about stigma, PLWH may hesitate to report psychological symptoms; thus, interventions and treatment for the relatively easy-to-report symptom of sleep disturbances may be more feasible than those for depression or anxiety.

We further found that a longer time since HIV diagnosis was associated with a statistically significant increase in the prevalence of sleep disturbances after adjusting for mean age. In recent years, advancements in ART and optimised virus control have led to a reduction in sleep disturbances previously associated with immunosuppression, opportunistic infection, and ART side effects, as observed in the 1990s^35,36^. However, the significant increase in sleep disturbances along the duration of HIV infection, despite good virus control and reduced ART side effects, suggests a potential influence of social and psychological factors. A lack of social support^14^ or low socioeconomic status^36^ may contribute to the sustained impact of sleep disturbances. Additionally, the internalised stigma PLWH experience due to their HIV status may be associated with depression and anxiety^37,38^. However, from the 20 studies that reported on the time since HIV diagnosis, the overall proportion of patients who were diagnosed with HIV over 5 years before was greater than 50%. Considering that previous studies have observed higher rates of sleep disturbances in the months soon after HIV diagnosis^5,39,40^, our findings should be interpreted with caution.

We also explored the impact of mean CD4 cell counts on the prevalence of sleep disturbances but found no significant association in our results. Previous findings have shown that lower HIV RNA levels were associated with higher sleep efficiency and revealed no relationship between lower CD4 cell counts and greater sleep disturbances^41^, which, together with our findings, suggests that HIV viral control may have a more significant impact on sleep disturbances than the CD4 cell count. However, given the scarcity of studies reporting HIV RNA levels in our review, we could not assess its relationship with the prevalence of sleep disturbances. Additional future analyses on this issue are necessary.

Furthermore, sleep disturbances include a wide range of disorders including insomnia, sleep-disordered breathing, hypersomnolence, circadian rhythm sleep–wake disorders, sleep-related movement disorders and parasomnias^42^. There are reports of higher prevalence of REM sleep behavior disorder and nocturia among PLWH^43^, as well as increased prevalence of obstructive sleep apnea^44^, indicating that sleep disturbances in this population extend beyond insomnia. Several pathophysiological changes suggest that HIV infection can cause sleep–wake dysregulation through early-stage immunological changes and sleep-promoting cytokines, while chronic immune activation and antiretroviral therapy side effects further disrupt sleep homeostasis^11^. Yet, the current review focused only on disturbances assessed via self-report measures, mainly evaluating insomnia. Therefore, additional research into the range of sleep disturbance disorders and their association with moderating factors is necessary.

In addition, social determinants of health, such as socioeconomic status (SES), are directly linked to the prevalence and incidence of sleep disturbances^45,46^. It has also been reported that socioeconomic determinants, including income, area of residence, and particularly educational attainment, are associated with HIV incidence^47^. Therefore, while our findings did not explicitly address various social determinants, it is also necessary to examine social determinants of sleep disturbances in PLWH. For instance, a study included in this meta-analysis also found that poor sleep quality in PLWH is significantly influenced by low monthly incomes and poor social support^48^. This suggests that addressing not only the depression, anxiety, and longer times since HIV diagnosis associated with sleep disturbances found in our study but also SES and other social determinants is beneficial for improving sleep health in PLWH.

The major strength of our study is that it is the most comprehensive systematic review to date on the prevalence of self-reported sleep disturbances in PLWH. More than half of the included studies were published in the last 5 years, allowing for a better reflection of recent trends. We also incorporated a detailed exploration of regional differences. Furthermore, we considered depression and anxiety as psychological moderators, which the previous meta-analysis did not take into account. Our study also has several limitations. First, high heterogeneity was observed in the meta-analysis, as anticipated from psychiatric research using subjective self-reported measurements. Second, most included studies were cross-sectional, cohort, and case–control studies, raising the possibility of selection bias due to the absence of randomisation and complicating the validation of the direction of causality between sleep disturbances and moderators in PLWH. Third, the inclusion of only English studies and the omission of grey literature resulted in publication bias. Fourth, we did not incorporate prevalence data derived from objective measures of sleep, such as polysomnography and actigraphy. Fourth, it is difficult to generalise the differences in the prevalence of sleep disturbances between continents because the included studies were relatively localised rather than being conducted in diverse countries. Lastly, we did not account for the impact of ART, which could induce sleep disturbances.

## Conclusion

This systematic review and meta-analysis demonstrated the pooled prevalence of self-reported sleep disturbances in PLWH, which was higher than that in the general population. Given the shift in focus from extending life expectancy to improving quality of life among PLWH, there is a need for effective evaluation and management of sleep disturbances in this population. Moreover, the study region, depression and anxiety comorbidity rates, time since HIV diagnosis, and sleep measurement tool used may be significant moderators of the prevalence of sleep disturbances in PLWH. These factors may therefore be useful in designing tailored screening programmes for high-risk patients and intervening early to prevent the onset and exacerbation of sleep disturbances in PLWH.

## Methods

### Design

The systematic review and meta-analysis were conducted in accordance with the Cochrane Handbook for Systematic Reviews of Interventions^49^ and Preferred Reporting Items for Systematic Reviews and Meta-Analyses guidelines^50^. The review protocol was prospectively registered in the PROSPERO database (registration number CRD42023468139).

### Data source and search strategy

The search for eligible studies was conducted using three different databases—PubMed, PsycINFO, and EMBASE—from the inception of each database to May 16, 2023. We used the following search terms to search titles and abstracts: (“sleep*” OR “insomnia”) AND (“HIV” OR “AIDS”). Lists of the articles detected through the search were downloaded, stored, and reviewed using EndNote (version 21.2).

### Study selection criteria

To be eligible for inclusion in the present meta-analysis, a study had to meet the following criteria: (1) participants were adult patients (≥ 18 years of age) with HIV infection (either tested or self-reported), (2) presented self-reported frequency data on sleep disturbances (including any type or diagnostic criteria) or relevant data that could be used to estimate the prevalence of sleep disturbances, (3) observational study design, including cross-sectional, cohort, case–control or longitudinal (with baseline data) studies, and (4) published in English. Studies were excluded if they included only participants with sleep disturbance at the time of enrolment or if they reported only objective measures for sleep disturbances or single-item measures with binary response options. When duplicate data were found in multiple studies in the same database, only the study with the more comprehensive data was included for analysis.

### Data extraction and quality assessment

Two independent investigators (SAL and JWO) conducted data extraction. Any discrepancies detected were resolved by consensus. The details collected included the first author, year of publication, study design, country in which the study was conducted, mean age of participants, percentage of male participants, number of HIV-positive participants, measure of sleep disturbances, measures of depressive and anxiety symptoms with relative cut-off scores, CD4 cell counts, mean time since HIV diagnosis in years, and prevalence of self-reported sleep disturbances.

For the current systematic review and meta-analysis, the quality of the included studies was evaluated using the Joanna Briggs Institute’s critical appraisal tool, the Checklist for Prevalence Studies^51,52^. Two authors (SAL and JWO) again independently evaluated each of the included studies. Any discrepancies detected were resolved by reaching a consensus through discussion. The checklist consists of nine items with four possible answers—“yes”, “no”, “unclear”, and “not applicable”—for assessing the appropriateness of the sample frame, sampling method, sample size, statistical analysis, response rate, and description of study subjects and settings as well as the sufficiency of the data analysis, validity of the methods, and reliability of the measurements. In this study, we calculated a quality score for each study by assigning 1 point to each item marked “yes” in the assessment and 0 points for all other responses. We summed the points for each study, and the possible scores ranged from 0 to 9. Based on this quality score, studies were classified into three groups: low quality (scores 0–2), moderate quality (scores 3–6), and high quality (scores 7–9).

### Data analysis

All data analyses and visualisations were conducted with R (version 4.3.1) using the *meta* and *metafor* packages. Prevalence data were converted using a Freeman–Tukey double-arcsine transformation. The overall prevalence rates of self-reported sleep disturbances with corresponding 95% confidence intervals (CIs) were calculated with a random effects model in the HIV-infected population. The *I*^*2*^ statistic was used to assess the heterogeneity of the studies, with values above 50% representing high heterogeneity^53^. A Baujat plot was employed to further investigate heterogeneity^54^; the plot’s x-axis depicted the contribution of each study to the overall heterogeneity statistic, and the y-axis represented the standardised difference in the total prevalence of sleep disturbances both with and without each study, indicating the impact of each study on the overall treatment effect. A sensitivity analysis was performed to assess the robustness of the primary results by excluding individual studies through the leave-one-out method. The statistical significance level was defined as *p* < 0.05 (two-tailed).

Subgroup and meta-regression analyses were conducted to investigate potential sources of heterogeneity. Subgroup analyses were performed for the continents (North America, South America, Africa, Asia, and Europe) and sleep instruments (Pittsburgh Sleep Quality Index [PSQI], Insomnia Severity Index [ISI], and other scales) categorical variables when there were at least two study outcomes in each subgroup. To assess if there were differences between studies published in 2015 or later compared to studies published before 2015, we also conducted subgroup analysis based on publication year. Meta-regression analyses were performed for the following continuous variables: mean age (years), percentage of participants with depression and anxiety over the relative cut-off scores, mean CD4 cell counts, and mean time since HIV diagnosis (years). To account for the relationship between age and sleep deterioration^55^, we also conducted multivariable meta-regression analyses for the percentage of participants with depression and anxiety over the relative cut-off scores, mean CD4 cell counts, and mean time since HIV diagnosis (years) after adjusting for mean age. The studies with missing covariate information were excluded from the meta-regression. Publication bias was evaluated using a funnel plot, Egger’s test, and a trim-and-fill analysis^56^.

### Ethics declarations

As this is a secondary literature-based study, ethical approval is not necessary.

### Supplementary Information

Supplementary Information.

## Data Availability

All data generated or analysed in this study are included in this article and its supplementary information files. Aggregated data can be shared by the corresponding author on reasonable request.
