# Supplementary material for: The prevalence and moderating factors of sleep disturbances in people living with HIV: a systematic review and meta-analysis
Source: Sci Rep. 2024 Jun 27;14:14817. doi: 10.1038/s41598-024-65713-x (PMC11211430; doi:10.1038/s41598-024-65713-x)

**The prevalence and moderating factors of sleep disturbances in people living with HIV: A systematic review and meta-analysis**

Suonaa Lee^1+^, Jae Won Oh^2+^, Kyung Mee Park^1,3^, Jin Young Ahn^4^, San Lee^1,5*^, Eun Lee^1,6*^

^1^Department of Psychiatry and the Institute of Behavioral Science in Medicine, Yonsei University College of Medicine, Seoul, Republic of Korea

^2^Department of Psychology, The University of Utah Asia Campus, Incheon, Republic of Korea

^3^Department of Hospital Medicine, Yongin Severance Hospital, Yonsei University College of Medicine, Yongin, Republic of Korea

^4^Division of Infectious Diseases, Department of Internal Medicine and AIDS Research Institute, Yonsei University College of Medicine, Seoul, Republic of Korea

^5^Department of Psychiatry, Yongin Severance Hospital, Yonsei University College of Medicine, Yongin, Republic of Korea

^6^Institute for Innovation in Digital Healthcare, Yonsei University, Seoul, Republic of Korea

^+^Co-first authors of the manuscript. These authors contributed equally to this work.

* Corresponding authors:

San Lee, MD, MPH

Department of Psychiatry, Yongin Severance Hospital

363 Dongbaekjukjeon-daero, Giheung-gu, Yongin-si,

Gyeonggi-do 16995 Republic of Korea

Tel.: +82-31-5189-8531; Fax: +82-31-5189-8565

E-mail: sanlee@yonsei.ac.kr

Eun Lee, M.D., Ph.D.

Department of Psychiatry, Yonsei University College of Medicine

50-1 Yonsei-ro, Seodaemun-gu, Seoul 03722 Republic of Korea

Telephone: +82 2 2228 1620; Fax: +82 2 313 0891

Email: leeeun@yuhs.ac

**SUPPLEMENTARY MATERIALS**

**Table of Contents**

**Supplementary Table 1**. Quality assessment of included studies using the Joanna Institute (JBI) critical appraisal tool for prevalence studies

**Supplementary Figure 1**. The result of sensitivity analysis

**Supplementary Figure 2**. Baujat plot

**Supplementary Figure 3**. Bubble plot of impact of anxiety on prevalence of sleep disturbances

**Supplementary Figure 4**. The funnel plot of included studies

**Supplementary Figure 5**. The result of trim and fill analysis

Supplementary Table 1. Quality assessment of included studies using the Joanna Institute (JBI) critical appraisal tool for prevalence studies

| Study | 1. Was the sample frame appropriate to address the target  population? | 2. Were study participants sampled in an appropriate way? | 3. Was the sample size adequate? | 4. Were the study subjects and the setting described in detail? | 5. Was the data analysis conducted with sufficient coverage of the identified sample? | 6. Were valid methods used for the identification of the condition? | 7. Was the condition measured in a standard, reliable way for all participants? | 8. Was there appropriate statistical analysis? | 9. Was the response rate adequate, and if not, was the low response rate managed appropriately? | score |
| --- | --- | --- | --- | --- | --- | --- | --- | --- | --- | --- |
| D De Francesco et al. | yes | unclear | unclear | yes | yes | yes | yes | yes | yes | 7 |
| Jabbari et al. | yes | yes | unclear | yes | yes | yes | yes | yes | yes | 8 |
| Daubert et al. | yes | yes | unclear | yes | yes | yes | yes | yes | yes | 8 |
| Huang et al. | yes | yes | unclear | yes | yes | yes | yes | yes | yes | 8 |
| Arbune et al. | yes | yes | unclear | yes | yes | yes | yes | yes | yes | 8 |
| Ning et al. | yes | yes | yes | yes | yes | yes | yes | yes | yes | 9 |
| Mahboobi et al. | yes | no | unclear | yes | yes | yes | yes | yes | yes | 7 |
| Marion et al. | yes | unclear | unclear | yes | yes | yes | yes | yes | yes | 7 |
| Downing et al. | yes | no | unclear | yes | yes | no | yes | yes | no | 5 |
| Ren et al. | yes | yes | unclear | yes | yes | no | yes | yes | yes | 7 |
| Chen et al. | yes | unclear | unclear | yes | yes | no | yes | yes | yes | 6 |
| Rubinstein et al. | yes | no | unclear | yes | yes | yes | yes | yes | yes | 7 |
| Redman et al. | yes | no | unclear | yes | yes | yes | yes | yes | yes | 7 |
| Rogers et al. | yes | no | unclear | yes | yes | yes | yes | yes | yes | 7 |
| Fekete et al. | yes | no | unclear | yes | yes | yes | yes | yes | yes | 7 |
| Robbins et al. | yes | no | unclear | yes | yes | yes | yes | yes | yes | 7 |
| Abdu et al. | yes | no | yes | yes | yes | yes | yes | yes | yes | 8 |
| Legas et al. | yes | yes | yes | yes | yes | yes | yes | yes | yes | 9 |
| Pujasari et al. | yes | no | yes | yes | yes | yes | yes | yes | yes | 8 |
| Crum-Cianflone et al. | no | no | unclear | yes | yes | yes | yes | yes | yes | 6 |
| Allavena et al. | yes | yes | unclear | yes | yes | yes | yes | yes | no | 7 |
| Cunha et al. | yes | yes | yes | yes | yes | yes | yes | yes | yes | 9 |
| Bedaso et al. | yes | yes | yes | yes | yes | yes | yes | yes | yes | 9 |
| Oshinaike et al. | yes | yes | unclear | yes | yes | yes | yes | yes | yes | 8 |
| Petrakis et al. | yes | unclear | unclear | no | yes | yes | yes | yes | yes | 6 |
| Chen et al. | yes | yes | yes | yes | yes | yes | yes | yes | yes | 9 |
| Seay et al. | yes | unclear | unclear | yes | yes | yes | yes | yes | yes | 7 |
| Kunisaki et al. | yes | yes | unclear | yes | yes | yes | yes | yes | yes | 8 |
| Faraut et al. | yes | unclear | unclear | yes | yes | yes | yes | yes | yes | 7 |
| Cruess et al. | yes | no | unclear | yes | unclear | yes | yes | yes | unclear | 5 |
| Pujasari et al. | yes | no | yes | no | yes | yes | yes | yes | yes | 7 |
| Najafi et al. | yes | unclear | unclear | yes | yes | yes | yes | yes | yes | 7 |
| Gutierrez et al. | yes | no | unclear | yes | yes | yes | yes | yes | yes | 7 |
| GebreEyesus et al. | yes | yes | yes | yes | yes | yes | yes | yes | yes | 9 |
| Phillips et al. | yes | unclear | unclear | yes | yes | yes | yes | yes | yes | 7 |
| Dabaghzadeh et al. | yes | unclear | unclear | yes | yes | yes | yes | yes | yes | 7 |
| Awopeju et al. | yes | yes | yes | yes | yes | yes | yes | yes | yes | 9 |
| Gamaldo et al. | yes | unclear | unclear | no | yes | yes | yes | yes | yes | 6 |
| Ding et al. | yes | yes | unclear | yes | yes | yes | yes | yes | yes | 8 |
| Lee et al. | yes | no | unclear | yes | yes | yes | yes | yes | yes | 7 |
| Junqueira et al. | yes | unclear | unclear | unclear | yes | yes | yes | yes | yes | 6 |
| Salahuddin et al. | yes | no | unclear | yes | yes | yes | yes | yes | yes | 7 |
| Byun et al. | yes | no | unclear | yes | yes | yes | yes | yes | yes | 7 |

Supplementary Figure 1. The result of sensitivity analysis


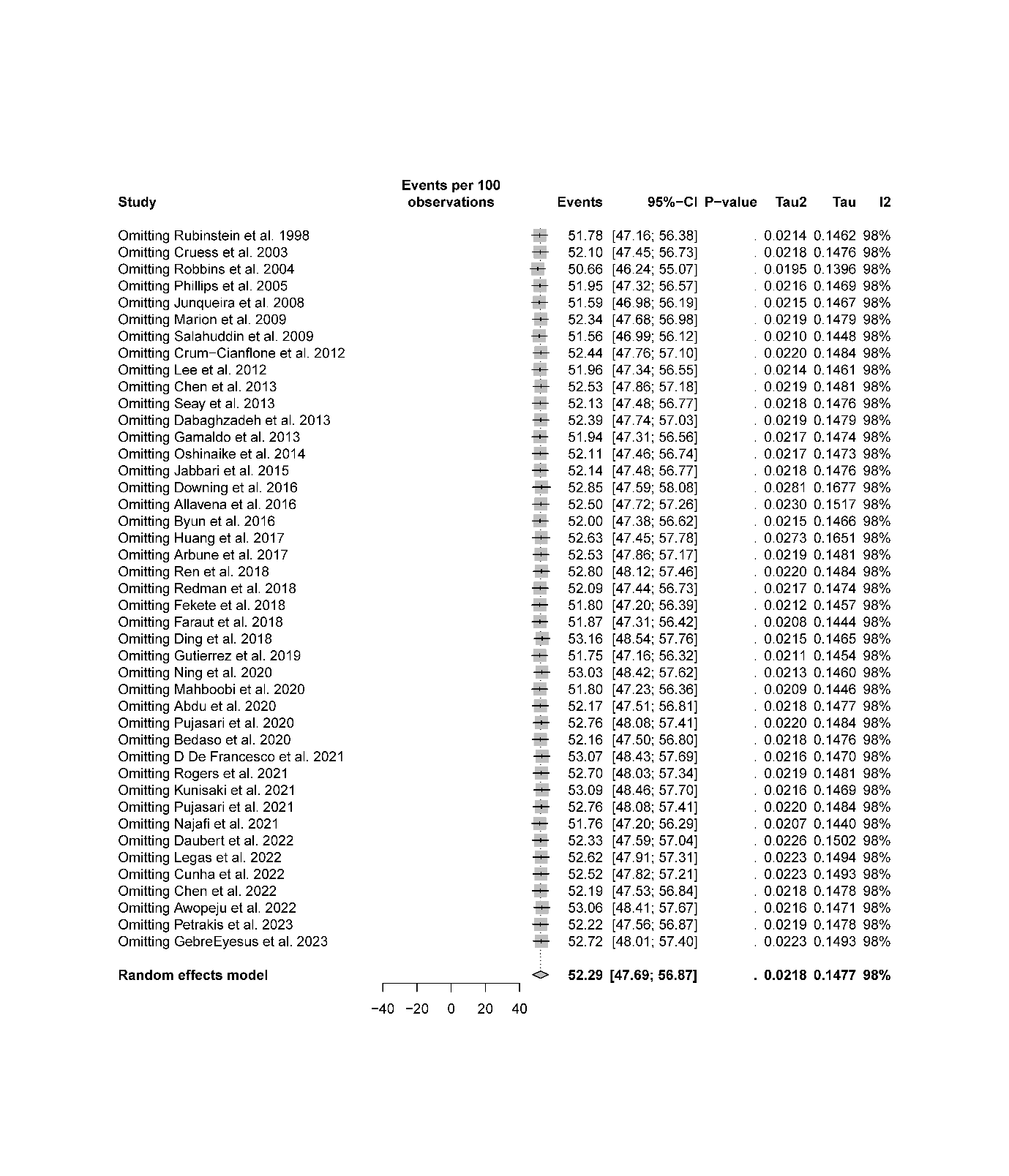


Supplementary Figure 2. Baujat plot


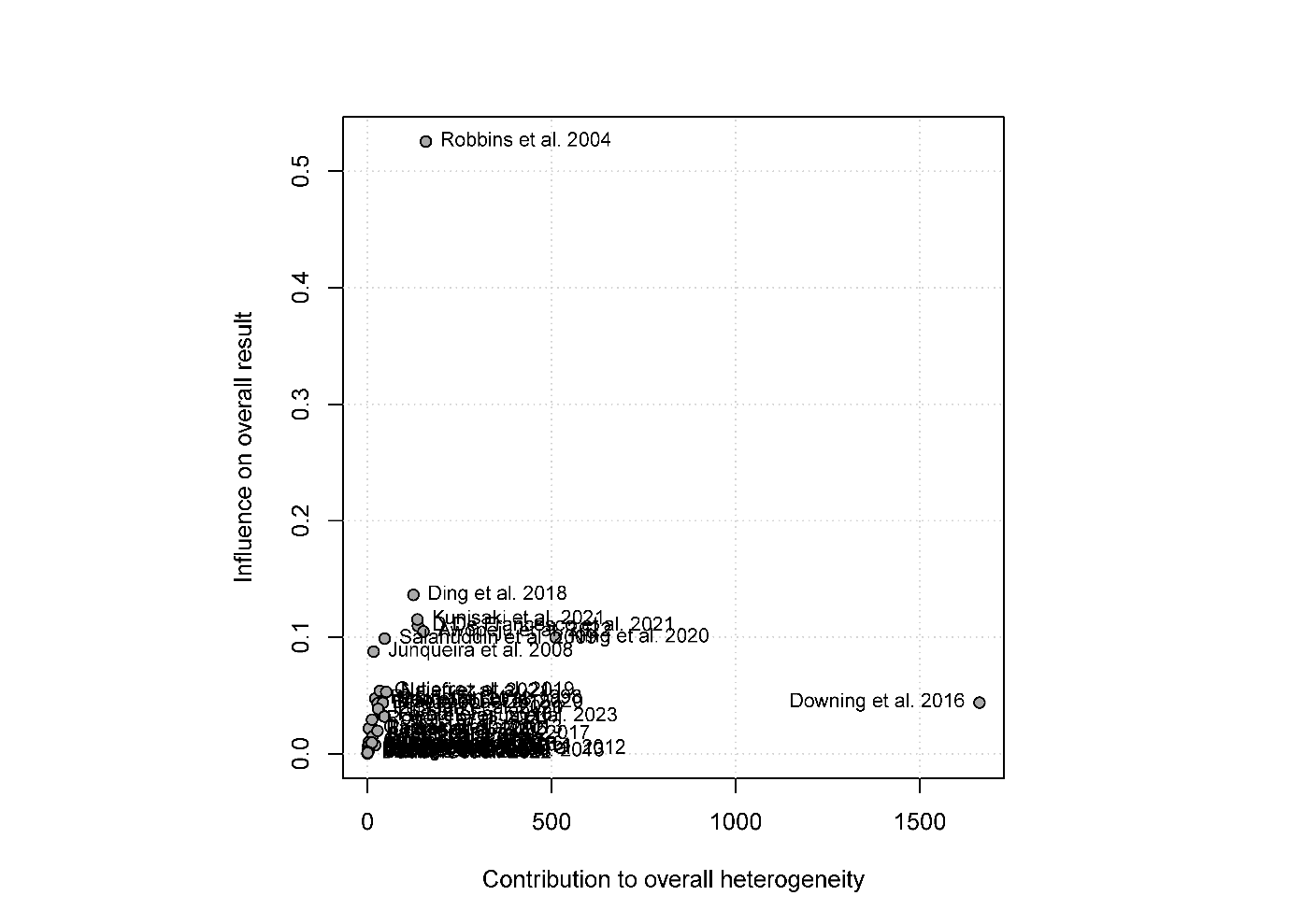


Supplementary Figure 3. Bubble plot of impact of anxiety on prevalence of sleep disturbances


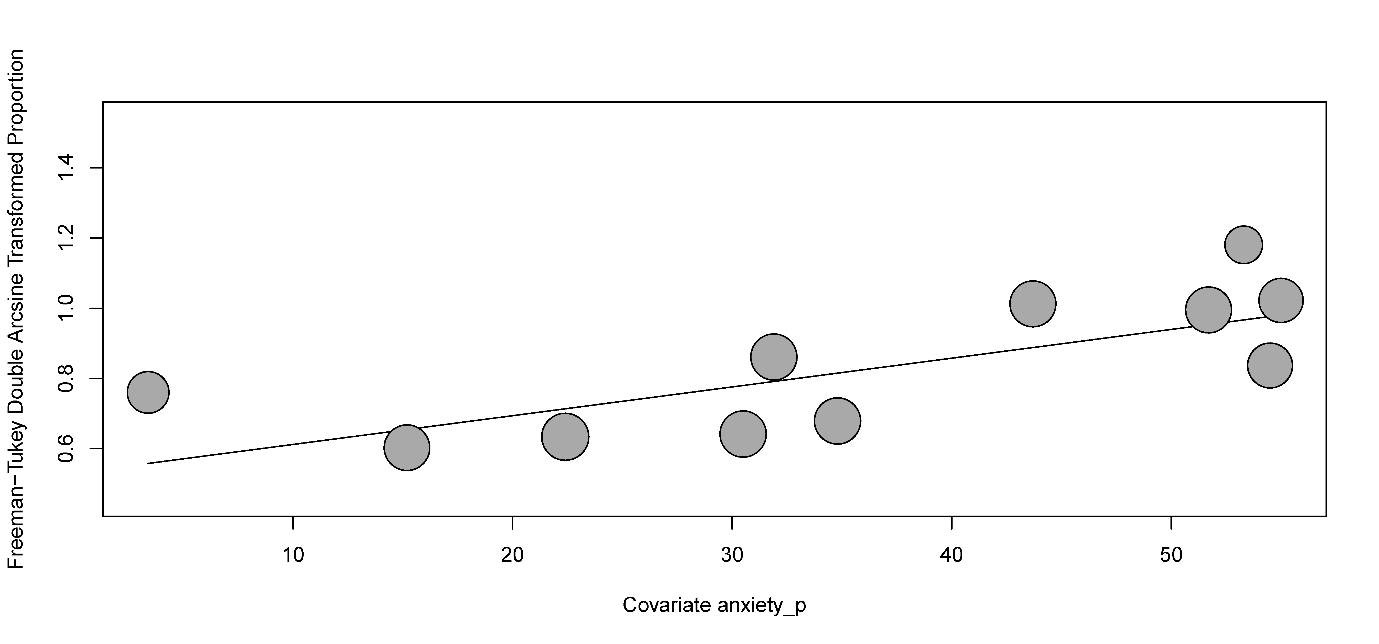


Supplementary Figure 4. The funnel plot of included studies


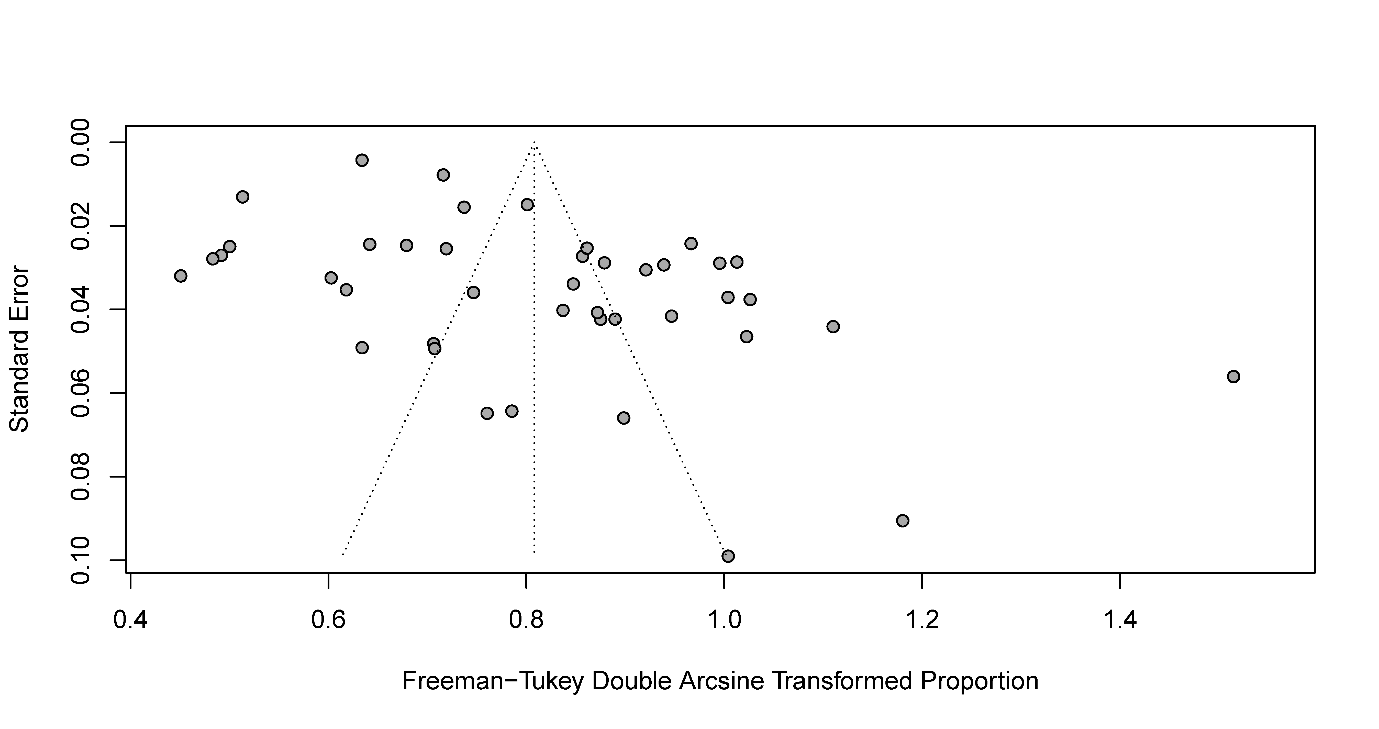


Supplementary Figure 5. The result of trim and fill analysis


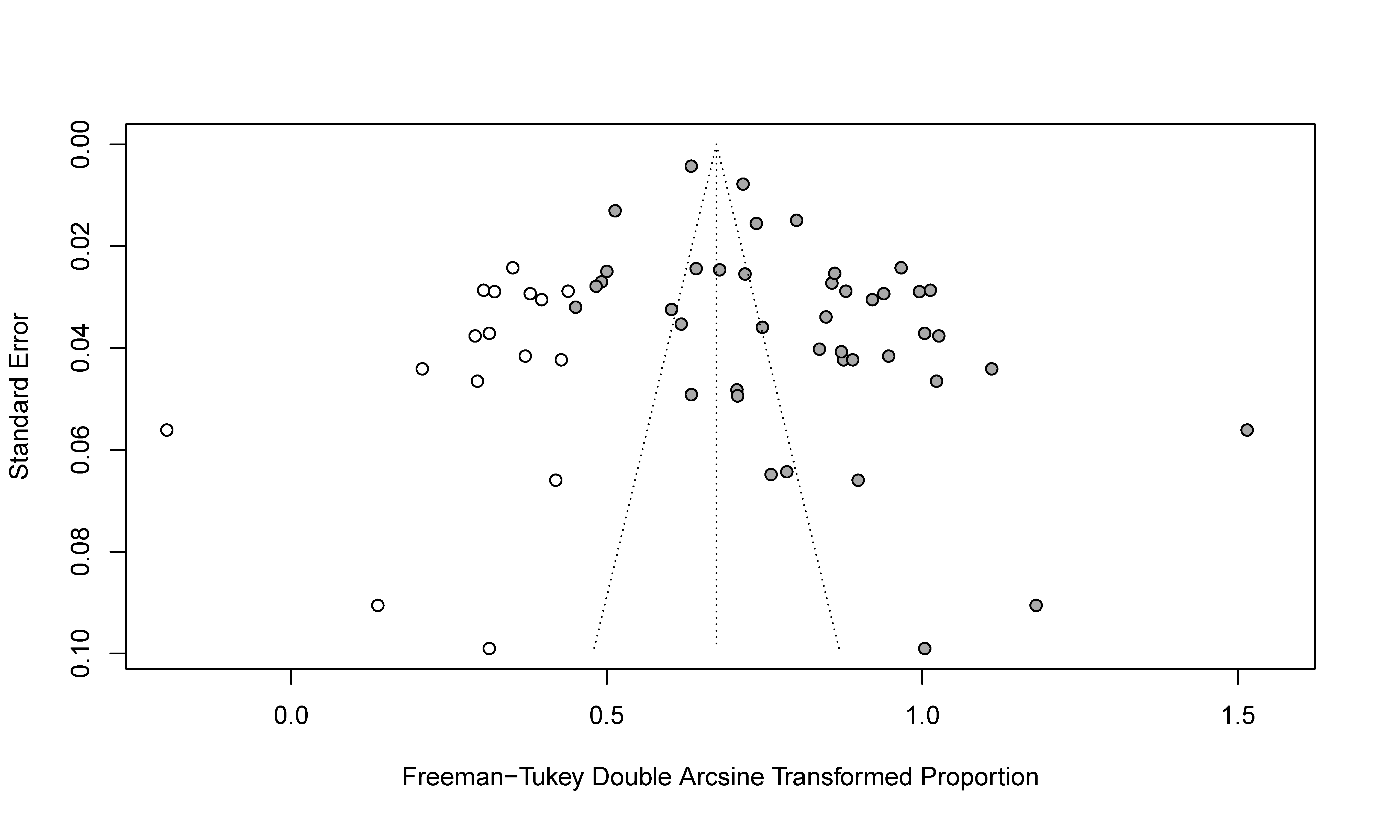

Supplement: Supplementary file 1 — Supplementary Information. [file 41598_2024_65713_MOESM1_ESM.docx]
